# Anti-Inflammatory and Anti-Arthritic Potential of Standardized Extract of *Clerodendrum serratum* (L.) Moon

**DOI:** 10.3389/fphar.2021.629607

**Published:** 2021-04-12

**Authors:** Raj Kumar Tiwari, Silpi Chanda, Udayabanu M, Manisha Singh, Shriya Agarwal

**Affiliations:** ^1^ Pharmacognosy and Phytochemistry, School of Health Sciences, Pharmaceutical Sciences, UPES, Dehradun, India; ^2^ Pharmacognosy and Phytochemistry, IEC School of Pharmacy, IEC University, Solan, India; ^3^ Pharmacology, Department of Biotechnology and Bioinformatics, Jaypee University of Information Technology, Solan, India; ^4^ Department of Biotechnology, Centre for Emerging Disease, Jaypee Institute of Information Technology, Noida, India

**Keywords:** anti-inflammatory, ursolic acid, standardization, HPTLC, arthritic, *Clerodendrum serratum*

## Abstract

**Aims:** Scientific biological evaluation of standardized extracts is becoming one of the central needs for the globalization of customary medication in current times. And to validate the presence of active constituents in crude medicinal extracts, analytical techniques like HPLC and HPTLC are the most suitable authentication systems. In the current study we aimed to standardize and evaluate *Clerodendrum serratum* (L.) Moon (Verbenaceae). For its unique anti-inflammatory and anti-arthritic properties. Evaluation and analysis of the plant, therefore, offers a new platform for the development of the herbal drug and could prove to be a safe and cost effective treatment for arthritis management.

**Methods:** The aqueous extract of C. serratum, a common plant in the Southeastern Asian region, was used for phytochemical investigation and standardization by HPTLC and HPLC. The standardized HPLC method was further validated by using ICH guidelines. The standardized extract was investigated for anti-inflammatory and anti-arthritic activity. Complete Freund’s adjuvant (CFA) model was performed to evaluate the activity. Paw diameter, joint diameter, arthritic score, and body weight was accepted as a parameter for the evaluation of biological activity.

**Results:** HPTLC method revealed the presence of ursolic acid with an R_f_ value of 0.38 and the amount quantified was 0.03% w/w. The presence of the bioactive phytochemical was further analyzed and confirmed by HPLC for which the validation was done successfully in accordance with ICH guidelines. The assay content for ursolic acid was found to be 0.059% with relative standard deviation (RSD) <2.5% for specificity and precision with spike recovery between 95–110%. The anti-arthritic activity of aqueous extract exhibited COX-2 and TNF-α inhibition as observed in various parameters like paw edema, arthritic index, and joint diameter. Plant extract showed reclamation of arthritis in regard to body weight, arthritic score, paw edema, and joint diameter. The extract showed significant results for TNF-α and COX-2(*p* < 0.0001). The plant extract also exhibited *in-vitro* anti-inflammatory activity.

**Conclusion:** The current study established the scientific basis of ethnomedicinal use of the plant for anti-inflammatory purposes and the management of arthritis and can also be used for quality control purposes.

## Introduction

Arthritis and its associated musculoskeletal imbalances is one of the common afflictions, affecting millions of people and greatly restricting their activities of daily living (Baranwal et al., 2012b). It is a common term used by medical practitioners to describe the progressive inflammatory responses to one or more joint structures due to various causes ranging from traumatic, rheumatic, to degenerative concerns resulting in muscular stiffness and restricted physical movements. Further, arthritis commonly affects persons from all age groups, race, sex, and global regions with more than 100 different types, such as juvenile arthritis, rheumatoid arthritis, ankylosing spondylitis, gout, psoriatic arthritis, and osteoarthritis, the last of which is degenerative. Its clinical features in suffering patients may vary from person to person and range from mild pain and swelling to intense forms like complete/partial joint immobility, muscular atrophy, and contractures (Rho et al., 2009). Subsequently, the drug regime includes administration of non-steroidal anti-inflammatory drugs (NSAIDs) to patients as a first line of treatment but its long term usage has led to certain potential adverse reactions, such as gastroduodenal diseases and renal insufficiency (Harirforoosh and Jamil, 2009), that are probably induced by the inhibition of cyclo-oxygenase for reduction in prostaglandin content. Although various types of treatments are available today, like NSAIDs, corticosteroids, and DMARDs, they are mainly used to treat the symptomatic concerns and do not target the pathological origin like membrane stabilizing, protein denaturation, etc. Moreover, treatment by the above available modes can cause serious hepatic damage, gastric bleeding (Craig and Cappelli, 2019), Hospitalization, and death (Rostom et al., 2005).

Therefore, to surpass all these issues and find a more harmless and equally efficacious therapeutic option, researchers are considering plants as a source of medicine. Initially, these plant-based medicinal systems have formed the foundation of folk or ethno medicines, practiced in India and some other parts of the world like China, Africa, and South America. Later a considerable part of this indigenous knowledge was formulated, documented, and eventually passed into organized systems of medicine such as Ayurveda, Unani Siddha, etc. Traditionally plants were used for various therapeutic claims by tribal communities throughout India (Jain and Singh, 2010; Balasankar et al., 2013; Nayak and Dhal, 2013). In our study we have selected the plant *Clerodendrum serratum* (L.) Moon belonging to the family *Verbenaceae*. This is a deciduous shrub widely distributed in the Western Ghats (Manjunatha, 2004) of India. In Ayurveda, the plant is popularly known as Bharangi (Sanskrit) and customarily called Blue Glory (English). As per the traditional claims, the roots of this plant are a potential source of medicine for ailments such as allergic disease, body soreness, respiratory illness, infectious disease, dropsy, eye diseases, fever, inflammation, malaria, opthalmia, rheumatism, snakebite, tuberculosis, ulcers, and wounds (Keshavamurthy, 1994). Studies reported various chemical constituents from the plant, like stigmasterol, bis(2-ethylhexyl) phthalate, hispulidin, serratumin A, acteoside, martynoside, serratumoside-A,myricoside, ursolic acid, spinasterol, spinasteryl-β-D-glucopyranoside etc., in various parts of plant including the stem, root, and erial part (Singh et al., 2012; Kumar and Nishteswar, 2013; Padal et al., 2013). The current study was focused on its *in-vitro* anti-inflamatory activity and *in-vivo* arthritis activity by using Complete Freund’s adjuvant (CFA) induced arthritis model.

## Materials and Methods

All the chemicals used in the current study were procured from Sigma Aldrich Co., St. Louis, United States. All other chemicals/solvent adopted were of analytical grade.

### Collection and Preparation of Extract

Roots of *C. serratum* L. were collected from Faizabad, Uttar Pradesh, India and taxonomical identification was done at the Department of Agronomy, Aacharya Narendra Dev Agriculture Technical University, Dist. Faizabad, Uttar Pradesh. The prepared brown colored powdered root (100 g) was extracted (decoction) with water (500 ml) at a temperature not exceeding 110°C for 2 h. The extract was strained and lyophilized. The yield of obtained dried extract was 10% w/w.

### Standardization of an Extract

#### Qualitative Estimation by High-Performance Thin Layer Chromatography

The presence of ursolic acid was identified by performing High-performance thin layer chromatography (HPTLC). The solvent system used for the analysis was toluene: ethyl acetate (8:2 v/v) as a binary mobile phase system. For spot detection, anisaldehyde sulfuric acid was used as the spraying reagent. Standard analytical grade of ursolic acid was used as a reference by dissolving in HPLC grade methanol. The obtained concentration was 1,070 µg/ml, which was further used for preparing the subsequent working standards. Camag Linomat V HPTLC system (Switzerland) equipped with 100 µl Camag syringe and scanner III was used for the current qualitative estimation. Sample and standard as narrow bands of a width of 3 mm were applied to precoated silica gel aluminum plate 60F-254.

#### Quantitative Estimation by High-Performance Thin Layer Chromatography

An isocratic HPLC method was developed and validated for the identification of ursolic acid. A prepacked column, C18 (25 cm × 4.6 mm) 5 µm, with UV detector (210 nm) was used for the current study. The injection volume was 20 µl and its flow rate was maintained at 0.6 ml/min. Run time for standard and sample were 45 min and the data acquisition was done. For HPLC analytical development studies, methanol and acetonitrile (30:70, v/v) were selected as the binary mobile phase system. Before using, it was filtered and degassed. Thereafter, the standard solution was prepared by adding 10 mg of ursolic acid in methanol (10 ml) and subjected for sonication. The solution was settled to room temperature and diluted further with the help of diluent up to the mark.

Then the sample solution was prepared by taking 1.5 g of the test sample in iodine flask and adding 25 ml of water followed by 20 min of sonication. Reflux was done for about 30 min in reflux assembly and filtered. The process was repeated twice and the filtrate was evaporated to dryness. The obtained residue was dissolved in methanol and the volume was made up of 10 ml methanol. A further 0.5 ml of this solution was taken into a 10 ml volumetric flask and diluted with methanol. Injection of equal volumes of the standard solution was done and chromatograms were recorded along with measurement of peak area. The percentage of ursolic acid was calculated by using the formula:

$$\%\;\text{of}\;\text{Assay}=\frac{\text{At}\;\text{XCsXP}}{\text{AsXCu}}$$

At= Peak area of a test sample, As = Peak area of reference standard, Cs = Concentration of reference standard, Cu = Concentration of test sample, P = Potency of ursolic acid working standard

The developed method was validated according to ICH guidelines (Baranwal et al., 2012a). The parameters adopted for its validation are Linearity, Specificity, Accuracy, Range, Precision, Repeatability, Intermediate precision, Robustness, Limit of Detection, and Limit of Quantification (Manukumar and Umesha, 2015).

### Evaluation of Anti-inflammatory

#### Hypotonic Solution-Induced Haemolysis or Membrane-Stabilizing Activity

The evaluation test was performed as per the method reported by Manukumar and Umesha (2015). The extracted sample contains 0.03 ml stock erythrocytes suspension infused with 5 ml hypotonic solution prepared with 154 mM NaCl in 10 mM Sodium Phosphate Buffer at pH 7.4, along with the test sample ranging from 100–500 μg/ml. The blank (without the test samples) and the standard (acetylsalicylic drug) was also treated accordingly. This mixture was incubated at room temperature for 10 min, followed by centrifugation for 10 min at 3,000 rpm. The absorbance of the supernatant was observed spectrophotometrically at 540 nm. The experiment was performed in triplicate. The percentage inhibition of haemolysis was computed by the following formula:

% inhibition  of haemolysis=100⋅[(A1−A2/A1)]

where: *A*1 = absorbance of blank; *A*2 = Absorbance of test and standard sample

#### Effect of Protein Denaturation

The effect of protein denaturation was performed as per the procedure mentioned below. The concentrations ranging from 100–500 μg/ml for both test sample and standard acetylsalicylic sample with 1 ml of 1 mM egg albumin solution were incubated at 27°C for 15 min. The mixture was further denatured at 70°C in a water bath for 10 min. The samples were allowed to cool down and observed spectrophotometrically at 660 nm. This experiment was performed in triplicate. Percentage inhibition of denaturation was calculated from the control without sample and standard (Badii and Howell, 2002; Chung et al., 2012). The percentage inhibition of denaturation was done by the below-mentioned formula:

% Inhibition of haemolysis=100X[(A1−A2/A1)]

### Evaluation of *In-Vivo* Anti-Arthritic Study

#### Complete Freund’s Adjuvant Induced Arthritis

For the *in-vivo* anti-arthritic activity, the Complete Freund’s Adjuvant (CFA) model was adopted. For this model, Albino Wistar male rats weighing 200 ± 25 g were considered. The oral route of administration was given to rats placed in different cage groups under a controlled temperature of 22 ± 2°C conditions with administration of golden feed diet and water to all the animals regularly. The dose adopted was 100 and 200 mg/kg body weight (b.w.). Paw diameter, joint diameter, arthritic score, and body weight were used as parameters for the activity. The histopathological estimation of TNF-α and COX-2 were also performed as confirmative studies. Institutional Animal Ethics Committee (IAEC) approval (Reg No. 1824/PO/ERe/S/15/CPCSEA) as well as Protocol Approval (Reference No. IAEC/PN-16045) were taken before performing the experiments.

In the current study, animals (6 nos.) were divided into five different groups. Group 1 received vehicle normal saline (10 ml/kg) orally before the 30 min waiting period. To group 2, 0.1 ml of CFA (0.05% *Mycobacterium butyricum* in mineral oil; 10 ml/kg b.w.) and vehicle (10 ml/kg) was injected into the left hind paw (subplantar surface) with the help of a 26 gauge needle. Group 3 received CFA and indomethacin (3 mg/kg b.w.) whereas group 4 and 5 administered samples at a dose of 100 mg/kg b.w. and 200 mg/kg b.w. respectively. For the arthritic evaluations, the paw diameter, joint diameter, arthritic score, and body weight measurements were carried out on the third, seventh, 14th, and 21st days. After completion of the protocol on the 21st day, the blood (terminal) samples were collected and the inflammatory mediators, like TNF-α and COX-2 levels, were estimated from the collected serum using ELISA kit assay for all the assigned groups. The ankle joint tissues were fixed with formalin and preserved for further histopathological studies. All obtained results were compared and evaluated with the standard (Pincus et al., 2003; Kamal and Baldi, 2015; Ghosh et al., 2016). The statistical analysis was subjected to mean ± Standard Error Mean (SEM). Differences were considered significant at *p* < 0.001, or *p* < 0.01, or *p* < 0.05 when compared test group vs. control (−ve) group. For numerical results, a one-way analysis of variance (ANOVA) (compare all vs. control) was performed using Graph Pad InStat Version 3 (GraphPad Software).

## Results

### Qualitative Estimation by High-Performance Thin Layer Chromatography

**FIGURE 1:**
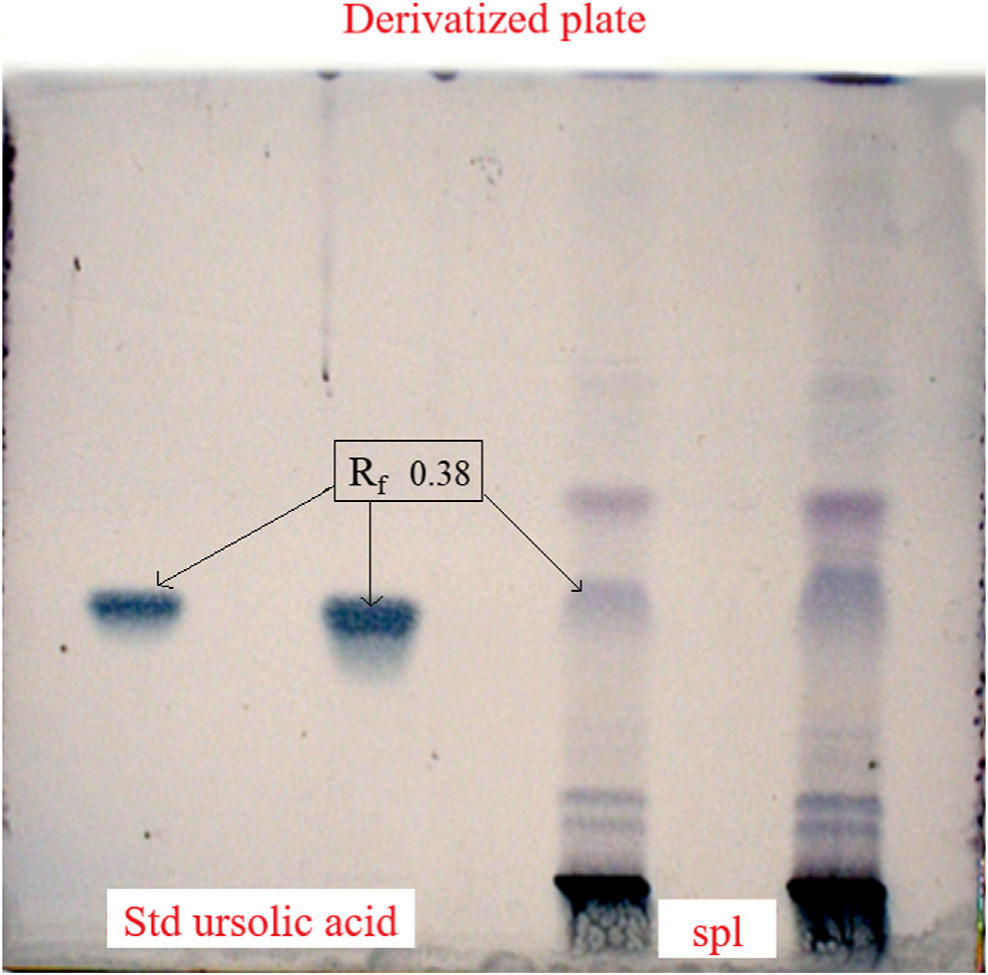
HPTLC chromatogram of ursolic acid standard and plant extract with R_f_ value.

**FIGURE 2:**
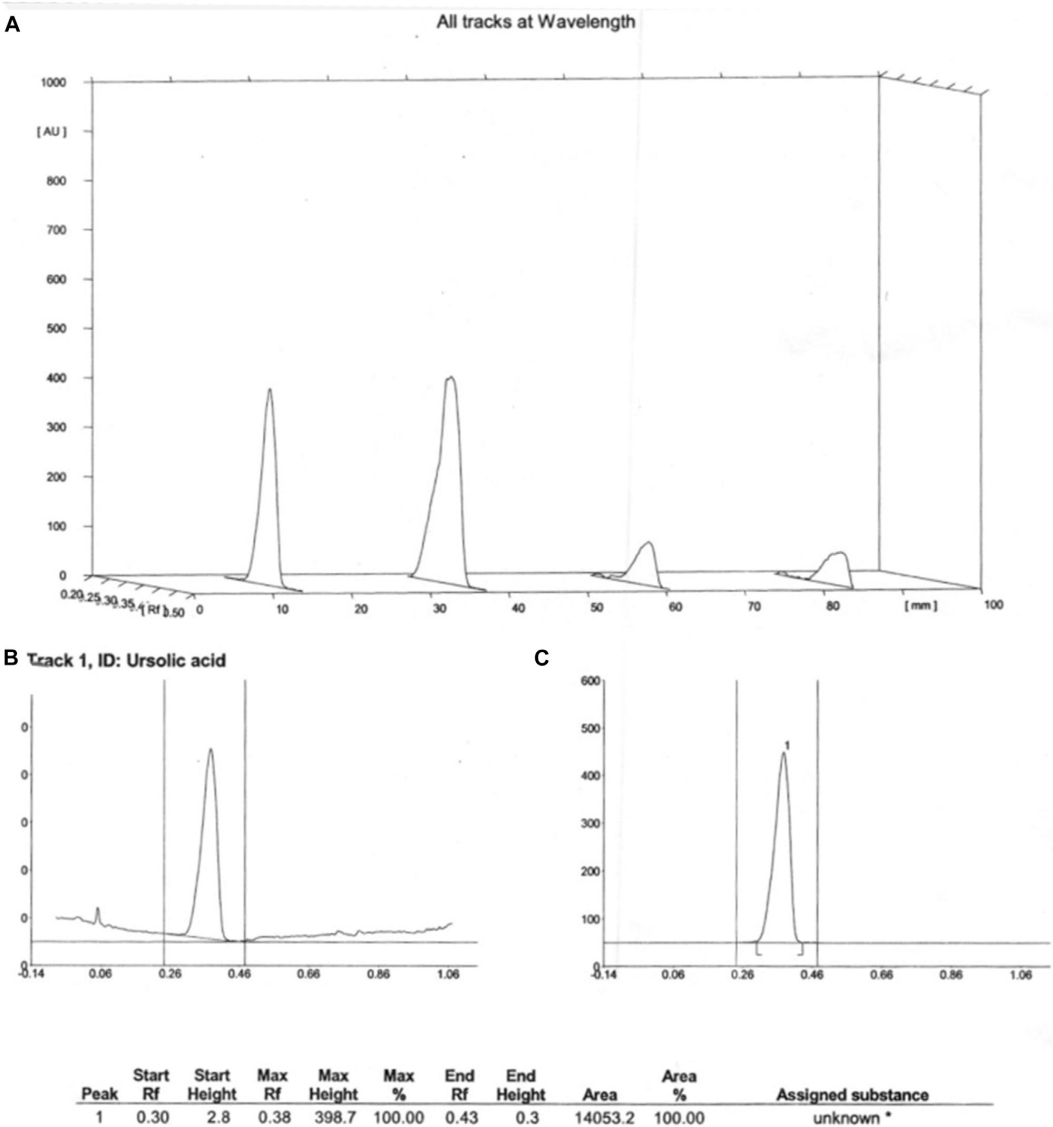
UV scanning chromatogram of plant extract **(A)** and ursolic acid standard **(B,C)**.

The presence of ursolic acid was confirmed by performing the HPTLC from the aqueous extract of *C. serratum* L. using standard ursolic acid. The stabilized HPTLC system produced a compact spot of standard ursolic acid with an R_f_ value of 0.38. HPTLC chromatogram of ursolic acid standard and plant extract are shown in Figure 1. UV scanning for sample (A) and standard (B,C) were graphically presented in Figures 2A–C.

### Quantitative Estimation by HPLC and Method Validation as per ICH Guidelines

**FIGURE 3:**
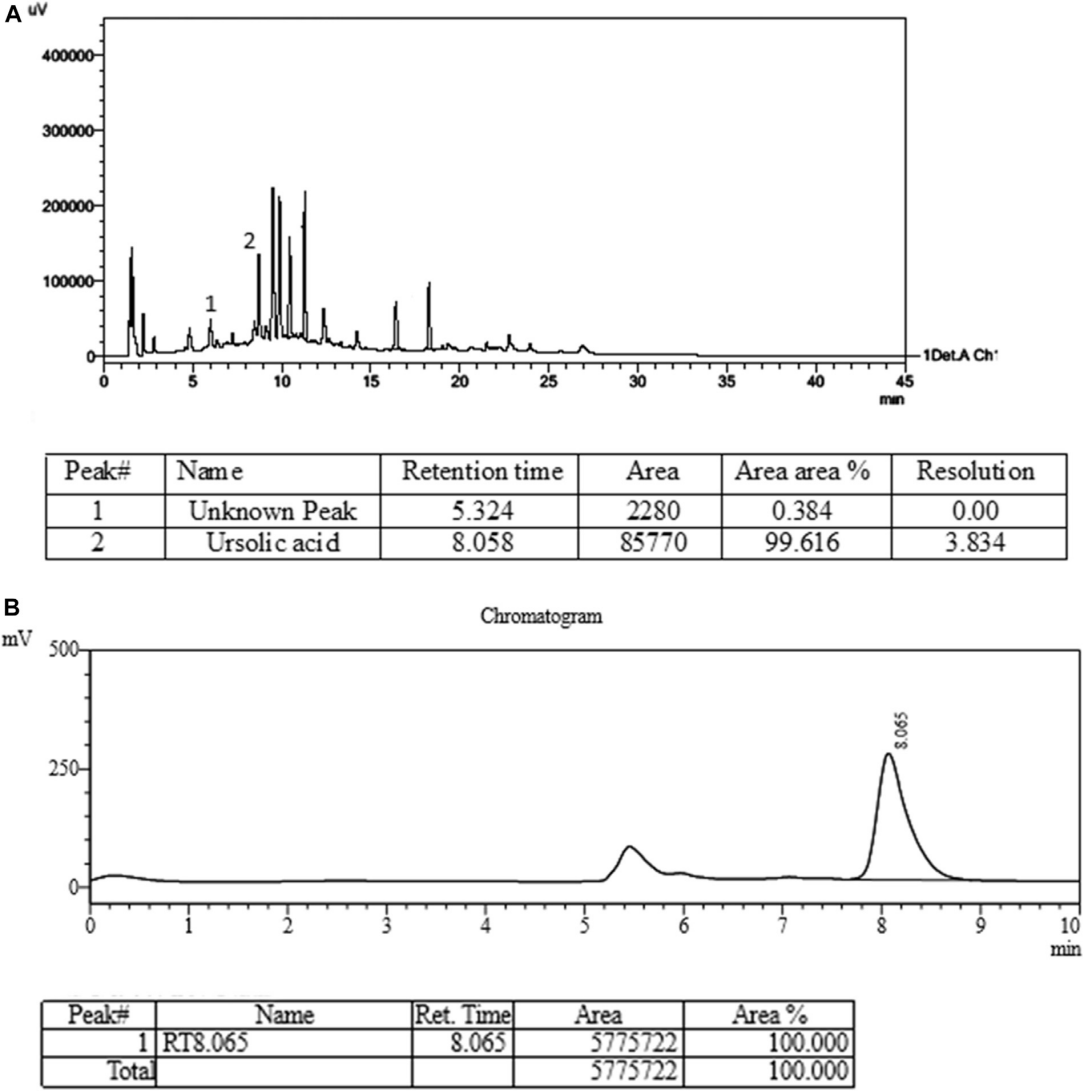
HPLC chromatogram of plant extract **(A)** and ursolic acid standard **(B)**.

The statistical analysis was performed by plotting the standard ursolic acid concentration and the peak responded with a straight line of correlation coefficient 0.998. The observed results showed good recovery within 95–110% when spiked with the standard sample at four different concentrations levels. The developed HPLC method was found to provide repeatable results at various concentrations; thus, the developed protocol was found to be reliable. The sample peak purity was confirmed by comparing with the standards of retention time and peak area. The LOD and LOQ were quantified at 33 µg/ml and 0.059% respectively for the ursolic acid of the test sample (extract). The performed analytical development and validation was assured as per the ICH guidelines and graphically presented in Figure 3 for sample (A) and standard (B).

### Evaluation of Anti-inflammatory and Anti-Arthritic

#### Hypotonic Solution–Induced Haemolysis or Membrane-Stabilizing Activity

**FIGURE 4:**
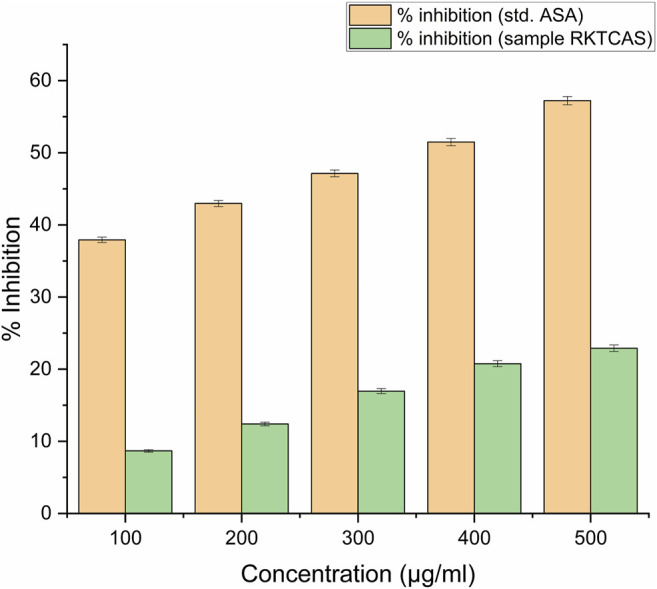
*In-vitro* membrane stabilizing activity showing the possible mechanism of action for the anti-inflammatory activity of aqueous extract of *Clerodendrum serratum* L.

Membrane stabilizing activity (8%) at a concentration level of 100 µg/ml was shown in Figure 4.

#### Effect of Protein Denaturation

**FIGURE 5:**
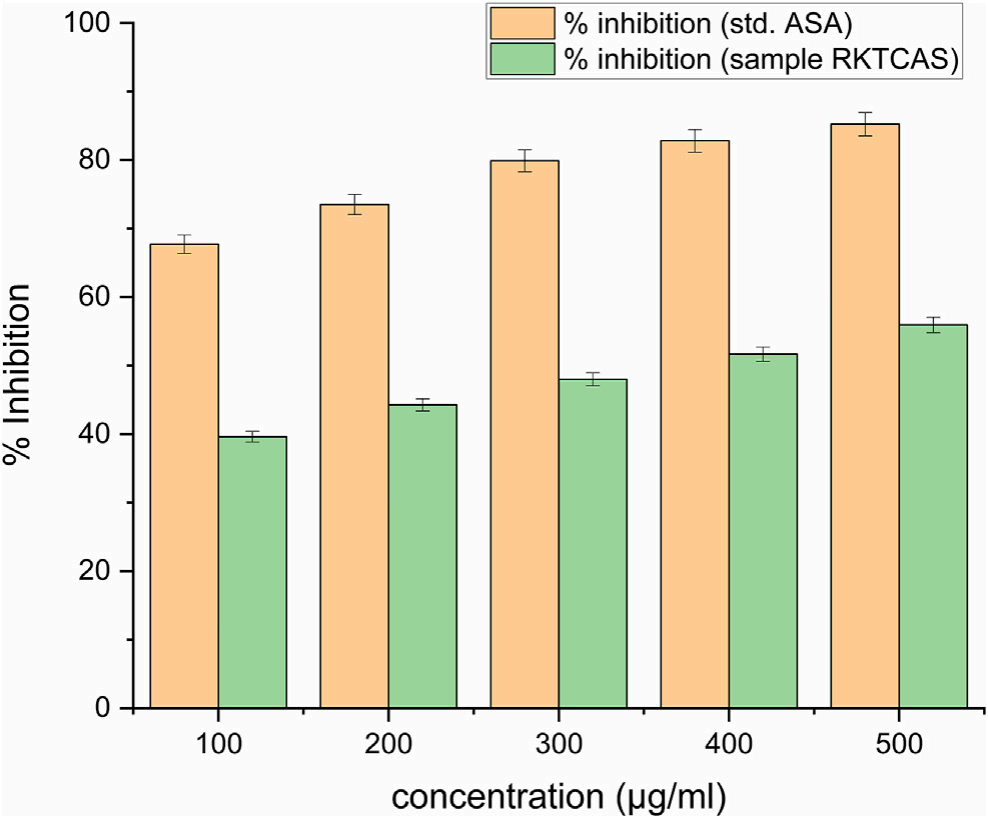
*In-vitro* protein denaturation activity of aqueous extract of *Clerodendrum serratum* L. against protein denaturation using egg albumin.

Around 40% of protein denaturation with 100 μg/ml concentration was shown in Figure 5.

### Evaluation of *In-Vivo* Anti-Arthritic Study

**FIGURE 6:**
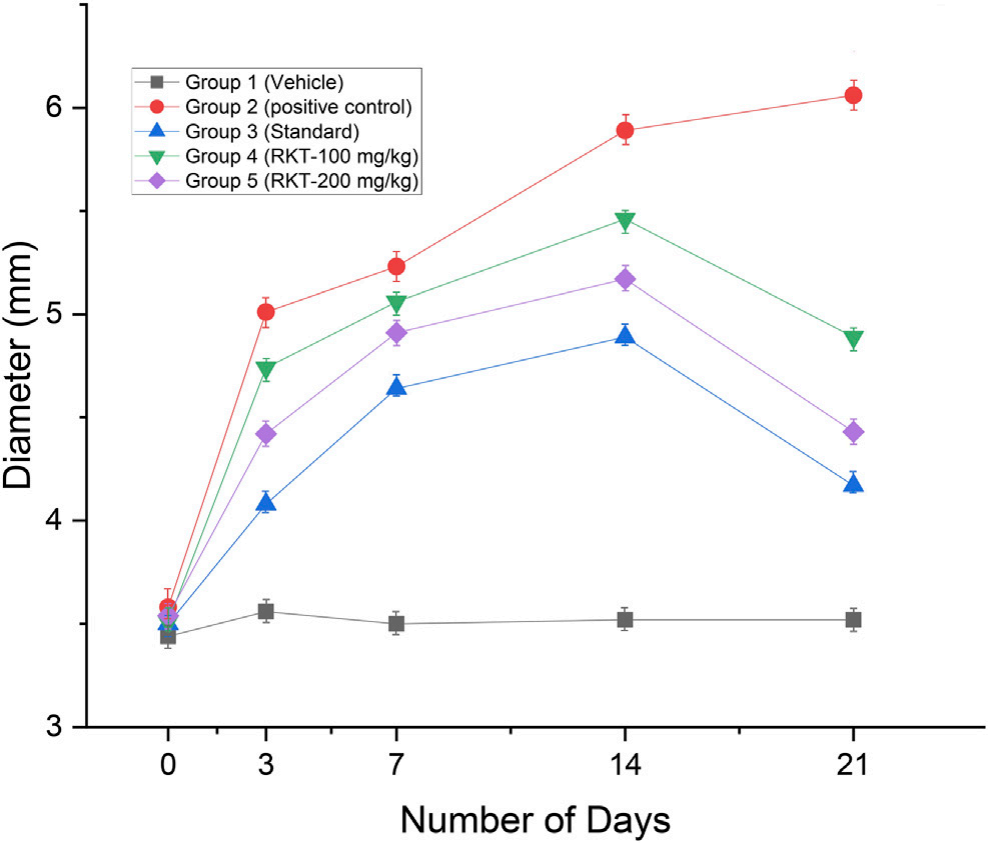
*In-vivo* anti-arthritic activity (Paw diameter in mm) *n* = 6; Data = Mean ± SEM; **p < 01 (G2 Vs G4); ***p < 0.001 (G2 vs. G3, G4 and G5).

**FIGURE 7:**
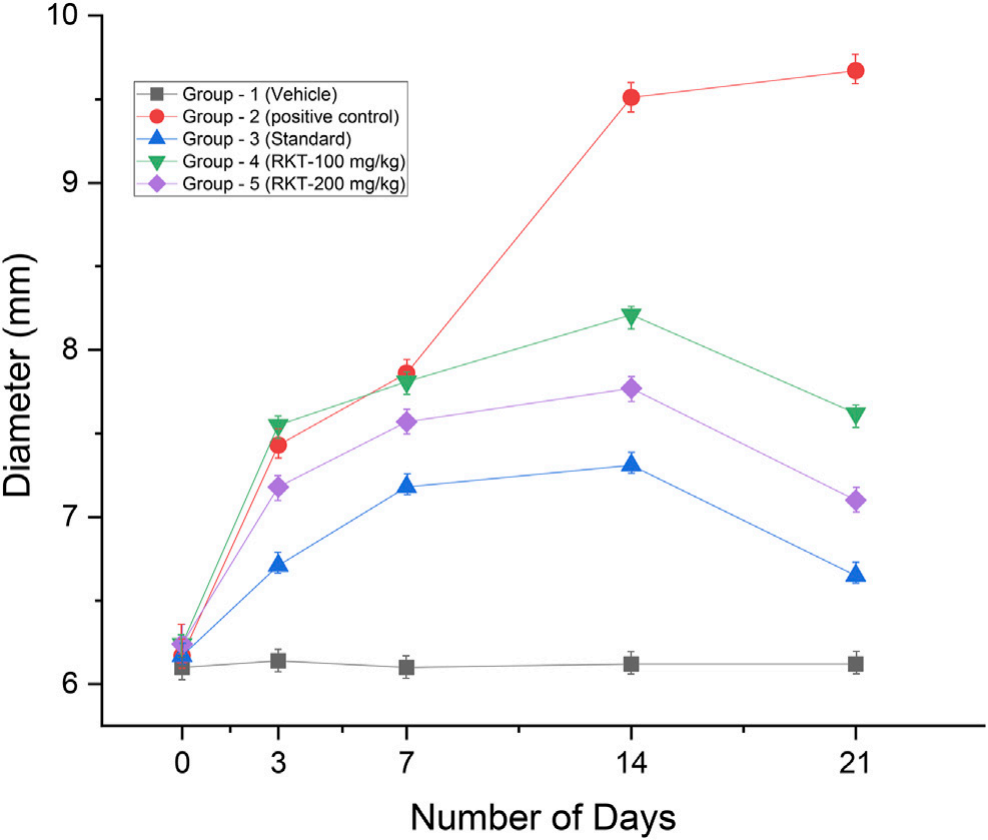
*In-vivo* anti-arthritic activity (joint diameter in mm). All the values are expressed as Mean ± SEM (*n* = 6) **p < 0.01 (G2 vs. G4); ***p < 0.001 (G2 vs. G3, G4 and G5).

**FIGURE 8:**
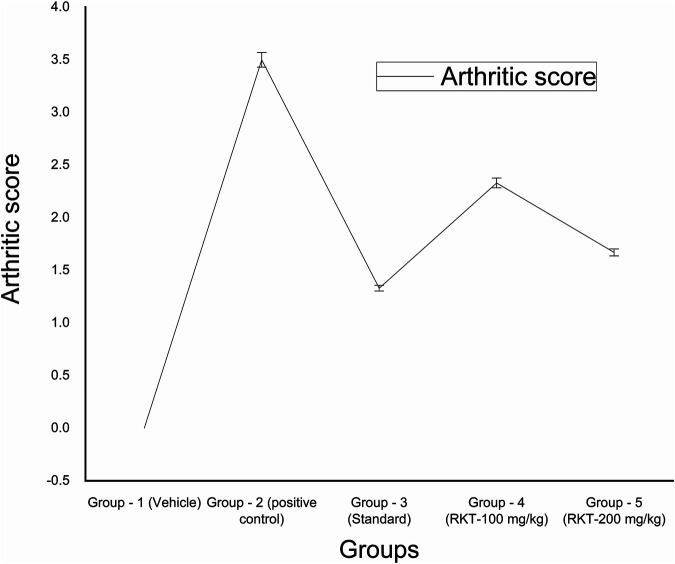
*In-vivo* anti-arthritic activity (arthritic score). All the values are expressed as Mean ± SEM (*n* = 6) *p < 0.05 (G2 vs. G4); ***p < 0.001 (G2 vs. G3 and G5).

**FIGURE 9:**
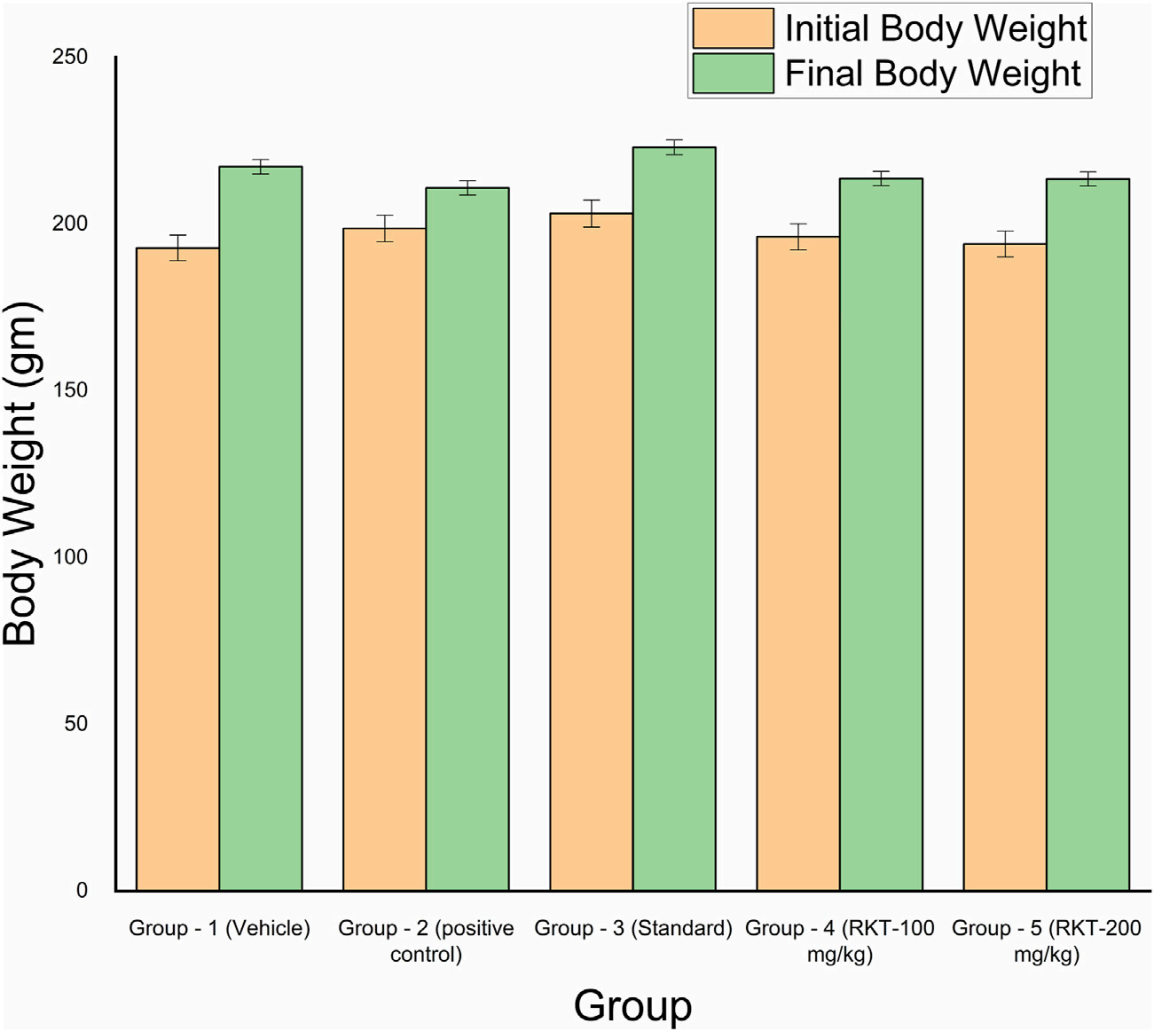
*In-vivo* anti-arthritic activity (bodyweight in gm).

**FIGURE 10:**
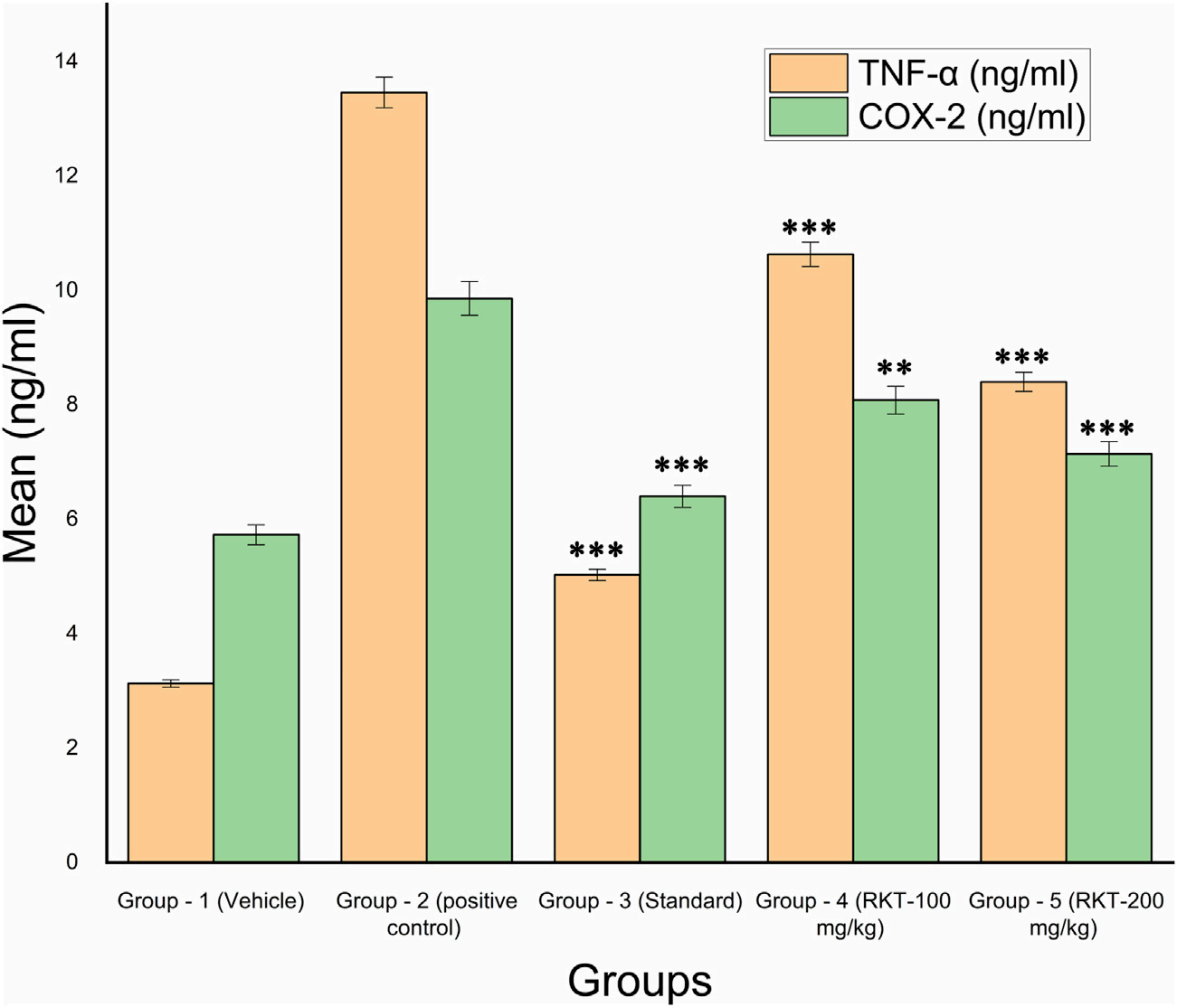
*In-vivo* TNF-α and COX-2 Estimation. All the values are expressed as Mean ± SEM (*n* = 6) ***p < 0.001 (G2 vs. G3, G4 and G5) For COX 2: **p < 0.01 (G2 vs. G4); ***p < 0.001 (G2 Vs G3 and G5).

**FIGURE 11:**
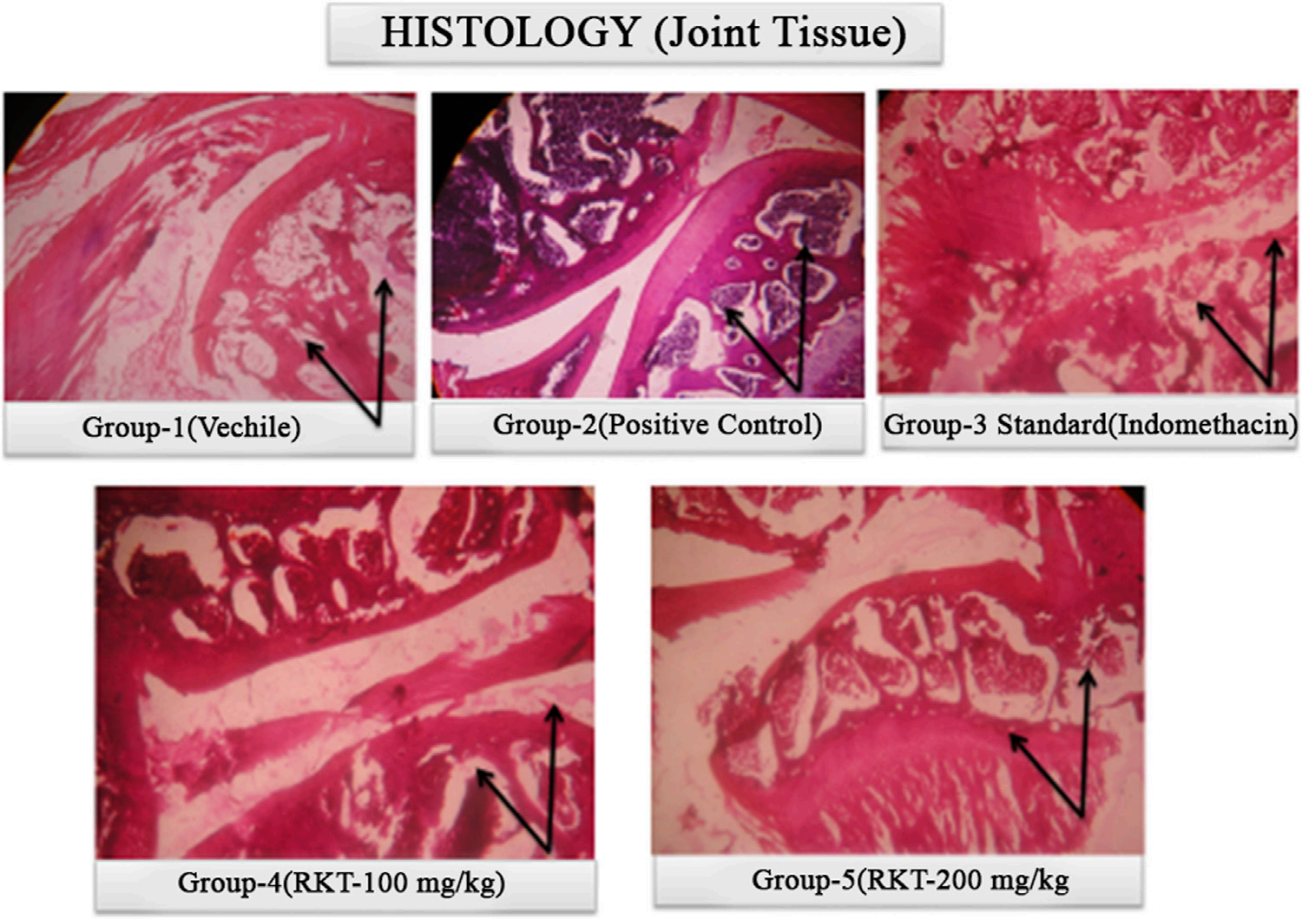
Histopathological study of ankle joint at 21 days of treatment after Complete Freund’s adjuvant (CFA) injection.

The measured arthritic parameters like paw diameter, joint diameter, arthritic score, and body weight were presented in Figures 6–9. Assessment of the blood sample was collected from the terminal parts of the animals. The level of TNF-α and COX-2 was estimated by using ELISA assay (Figure 10). The histopathological analysis of ankle joints after 21 days at a dose of 200 mg/kg was shown in Figure 11.

## Discussion

Arthritis is a persistent disease that equally affects adults and the elderly. Although the prevalence rate ranges between 0.3 and 1% (World Health Organization, 2020), women are mostly affected. As per Sharon et al., (2017), the high arthritis prevalence rate in low to middle-income countries leads to an inability to meet the daily personal as well as social needs. Fundamental endeavors toward prevention and management of arthritis should be prioritzed. The majority of the manufactured medicines, like NSAIDs (Ghosh et al., 2016), Glucocorticoids (Joseph et al., 2016), DMARDs (Pincus et al., 2003; Donahue et al., 2008; Luo et al., 2013; Kapoor et al., 2014) and certain biologicals (Banse et al., 2015; Brady et al., 2015; Zengin et al., 2017), target the symptoms but not the etiology of the disease with extreme adverse drug effects, whereas the surgical treatments (Krause and Matteson, 2014) can lead to post-operative complexities with mental unacceptance.

The natural product has a significant impact with fewer side effects and co-morbidities. Natural cures could be used to combat the constraints related with current accessible synthetic treatments. The plant Bharangi is a popular folk medicine used for inflammation, rheumatism, and pain. Specialists are considering utilizing the Bharangu plant to improve the symptoms of arthritis. The identification of metabolites with proper integration of modern scientific techniques in herbal research is an essential step for the global market and also for reproducible results. Thus, both HPLC and HPTLC analytical methods were used in the current study as the most applicable tools for identification and standardization.

In the current study, we focused on the root as a biological source in Ayurvedic Pharmacopoeia of India. Ursolic acid was identified and quantified by HPTLC and HPLC in the aqueous extract of the root. The developed HPLC method was also validated as per ICH guidelines. Shanmuga et al. (2017) likewise quantified the metabolites apigenin and luteolin by reverse phase high-performance liquid chromatography (RP-HPLC) from its leaf extract (Shanmuga et al., 2017). Followed by its analytical analysis, our study focused on its anti-inflammatory and anti-arthritic activity. In our study, it has been found that the presence of secondary metabolites enhances the stability of the biological membranes when stressed under lysis conditions, considered to be the preliminary investigation involved during the screening of anti-inflammatory activity. The RBC membrane structurally resembles the lysosomal membrane. The protein denaturation of the anti-arthritic property was successfully evaluated using *in-vitro* assessments. Literature (Krause and Matteson, 2014) also supported the use of ursolic acid in inhibition of the release of proinflammatory cytokines TNF-α, IL-2, and IFN-γ. Studies also revealed the presence of β-sitosterol as an anti-arthric potential in ethanolic extract of the leaf of the plant. The methanolic extract demonstrated dual inhibitory effects on arachidonic acid metabolism which is responsible for the inhibition of the synthesis of inflammatory mediators released through cyclooxygenase and lipoxygenase pathways (Cargnin and Gnoatto, 2017). Patel et al. (2014) revealed the presence of Apigenin-7-glucoside which further confirmed its anti-inflammatory effects and also claimed pain relief to knee osteoarthritis patients in a randomized controlled clinical trial study (Shoara et al., 2015). Thus the presence of phytochemicals like ursolic acid, flavonoid, apigenin, and β-sitosterol strongly support the traditional use and potentiality of *C. Serratum* as an anti-inflammatory and anti-arthritic.

## Conclusion

The current study showed that the extract possesses both anti-inflammatory and anti-arthritic activities. The possible mode of action of the anti-arthritic activity of the aqueous extract of *C. serratum* may be mediated by the inhibition of COX-2 and TNF-α. Based on the results, anti-arthritic activity may be due to shielding of a synovial membrane, obstruction of cartilage destruction, and inhibition of vascular permeability. Through our intensive efforts and thorough research, the results confirmed to show favorable arthritis restoration using arthritis parameters and histopathological observations from the selected plant *C. serratum.* The developed HPTLC and HPLC method could be used as an analytical tool for its quality control purpose.

## Data Availability

The original contributions presented in the study are included in the article/Supplementary Material, further inquiries can be directed to the corresponding author.
